# Primary Central Nervous System Lymphoma (PCNSL) Following Thyroid Cancer Surgery: A Case Report of Misdiagnosed Brain Metastasis and Literature Review

**DOI:** 10.3390/curroncol31120556

**Published:** 2024-11-26

**Authors:** Yilin Li, Tingyu Liang, Hao Xing, Yu Wang, Kuanyu Wang, Wenbin Ma

**Affiliations:** 1 Department of Neurosurgery, Center for Malignant Brain Tumors, National Glioma MDT Alliance, Peking Union Medical College Hospital, Chinese Academy of Medical Sciences and Peking Union Medical College, Beijing 100730, China; yilin_li@student.pumc.edu.cn (Y.L.); ltykobe0727@126.com (T.L.); xinghao1026@outlook.com (H.X.); 2 ‘4+4’ Medical Doctor Program, Chinese Academy of Medical Sciences and Peking Union Medical College, Beijing 100730, China; 3 Department of Stereotactic Radiosurgery, Beijing Tiantan Hospital, Capital Medical University, No. 119 South 4th Ring West Road, Fengtai District, Beijing 100070, China; wangkuanyu916@163.com

**Keywords:** primary central nervous system lymphoma, thyroid cancer, brain metastasis

## Abstract

**Objectives:** This article reports a rare case of primary central nervous system lymphoma (PCNSL) found in a patient with thyroid cancer after surgery. Methods: The patient was initially misdiagnosed with brain metastases, and the diagnosis of PCNSL was later confirmed by pathology. Results: The analysis of this case and review of the relevant literature explores the possible mechanisms of the coexistence of thyroid cancer and PCNSL, as well as their diagnostic, differential diagnostic, and therapeutic challenges. Conclusions: The article suggests a possible correlation between the coexistence of multiple cancers and autoimmune diseases and emphasizes that disease cannot be only considered in a monolithic way.

## 1. Introduction

Thyroid cancer is one of the most common endocrine malignancies, among which papillary thyroid carcinoma (PTC) is the most common, and the prognosis is usually good. Although central nervous system (CNS) metastases in differentiated thyroid cancer are rare, ranging from 0.3 to 5% [1], their prognosis is worse than that of patients without metastases, with a mean overall survival (OS) range of 7.1–33 months [2,3,4]. Brain metastases from thyroid cancer are less common, especially in papillary thyroid cancer. Brain metastases may be asymptomatic, but more often symptoms of brain metastases include neurological deficits, signs of increased intracranial pressure, neuropsychological symptoms, and seizures [5,6,7]. The interval between primary tumor diagnosis and identification of BM is quite long (101 months), and extracranial metastases are detected in about 95% of patients with thyroid cancer brain metastasis [8]. Brain metastasis (BM) is an important consideration when a patient with a history of thyroid cancer presents with neurologic symptoms. Its treatment includes surgical resection and radiotherapy [9].

Primary central nervous system lymphoma (PCNSL) is a rare and highly aggressive non-Hodgkin’s lymphoma that accounts for approximately 4% of all brain tumors and 4–6% of all extra-nodal lymphomas [10]. PCNSL can occur at any age, but the median age is 60 years. The incidence of PCNSL has increased in recent years, particularly in immunocompromised individuals, but it can also occur in immunocompetent patients [11]. The pathological features of PCNSL are predominantly diffuse large B-cell lymphoma (90%), and histologically the cells are usually highly proliferative, diffusely infiltrate the brain parenchyma, and show a vascular center growth pattern [12,13]. Most cases of PCNSL present with focal neurological deficits (50 to 70%), altered mental status (40 to 50%), or increased intracranial pressure (33%) [12]. The clinical presentation is often similar to other neurological disorders, including brain metastases, making the diagnosis particularly challenging, especially in patients with a history of systemic malignancy.

Imaging plays a key role in differentiating PCNSL from brain metastases from thyroid cancer. Typical MRI presentation of PCNSL is that the lesions may be multifocal or unifocal, are usually periventricular (60%), and affect the deeper structures of the brain, such as the corpus callosum, the deep white matter, and the basal ganglia. It appears isointense on T1-weighted images, isointense or hyperintense on T2 sequencing, and hyperintense on diffusion weighted imaging (DWI). In contrast to glioma or solid tumor metastases, PCNSL lesions tend to show mild edema relative to their size [12]. The standardized maximum uptake value (SUVmax) of 18F-FDG PET/CT for PCNSL ranged from 8.4 to 27.8 with a mean value of 18.1 (95% CI, 16.0–20.1), compared with 10.4 for inflammatory or malignant brain diseases [14,15]. The imaging presentation of brain metastases from thyroid cancer varies depending on the degree of differentiation of the tumor. Thyroid cancer metastases usually show hypointense on T1WI and hyperintense on T2WI, with the majority showing inhomogeneous enhancement. Seventy-five percent of thyroid cancer metastases are often associated with peripheral edema, and heterogeneous enhancement is seen in 63% of patients, while 60% of patients present with internal bleeding in their BMs. The strongest predictor of BM hemorrhage is tumor size [8]. Although imaging manifestations can suggest the possibility of PCNSL, its overlapping imaging features with other CNS tumors lead to diagnostic difficulties.

This case report describes a patient with papillary thyroid carcinoma who developed intracranial space-occupying lesions many years after thyroid surgery. It was initially suspected to be brain metastases, but it was diagnosed as PCNSL by postoperative pathology, demonstrating the complexity and diagnostic challenge of this case. This article focuses on the challenges of the diagnostic process and the possible mechanisms and discusses the management of this case in the context of a literature review.

## 2. Case Description

### 2.1. Basic Patient Information

A 55-year-old woman with a previous diagnosis of papillary thyroid cancer recently presented with new neurological symptoms. The patient had a history of tuberculosis diagnosed 11 years ago, which was cured. There was no history of other chronic diseases, and a history of drug and food allergies was denied.

### 2.2. Medical History

**Figure 1:**
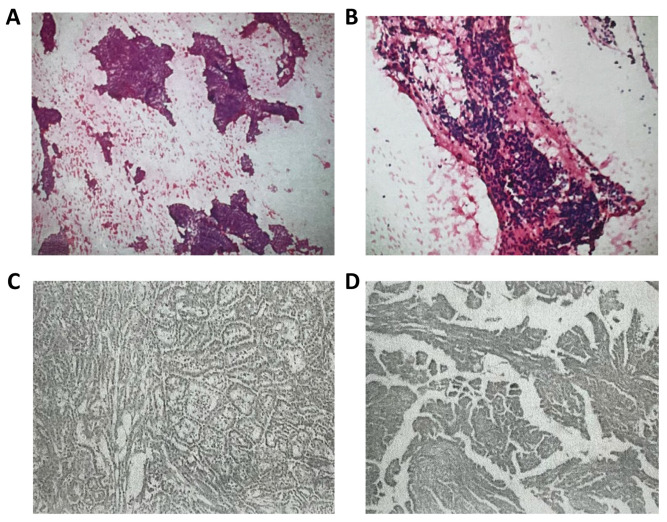
Thyroid pathology. (**A**,**B**) FNA; (**C**,**D**) thyroid isthmus and right lobectomy.

In December 2019, the patient was found to have thyroid isthmus and right-lobe nodules (BI-RADS grade 4A), with thyroid peroxidase antibody (TPOAb) 121.1 µ/mL (reference range 0–60), anti-thyroglobulin (TGAb) 178.4 µ/mL (reference range 0–60), and the rest were normal among the seven items of the thyroid workup. The fine-needle puncture (FNA) result suggested suspected papillary thyroid carcinoma, and subsequently thyroid isthmus and right lobectomy were performed in December 2019. Postoperative pathology suggested papillary thyroid carcinoma (T1N0M0) with BRAF V600E mutation (Figure 1). After the surgery, the patient took levothyroxine sodium tablets for thyroid hormone replacement therapy, and regular reexamination showed no obvious abnormality. Until April 2024, the patient had unfavorable movement of the left limb with dizziness, without nausea, vomiting, or seizures. The course of the patient’s diagnosis and treatment is shown in Table 1.

### 2.3. Diagnostic Process

**Figure 2:**
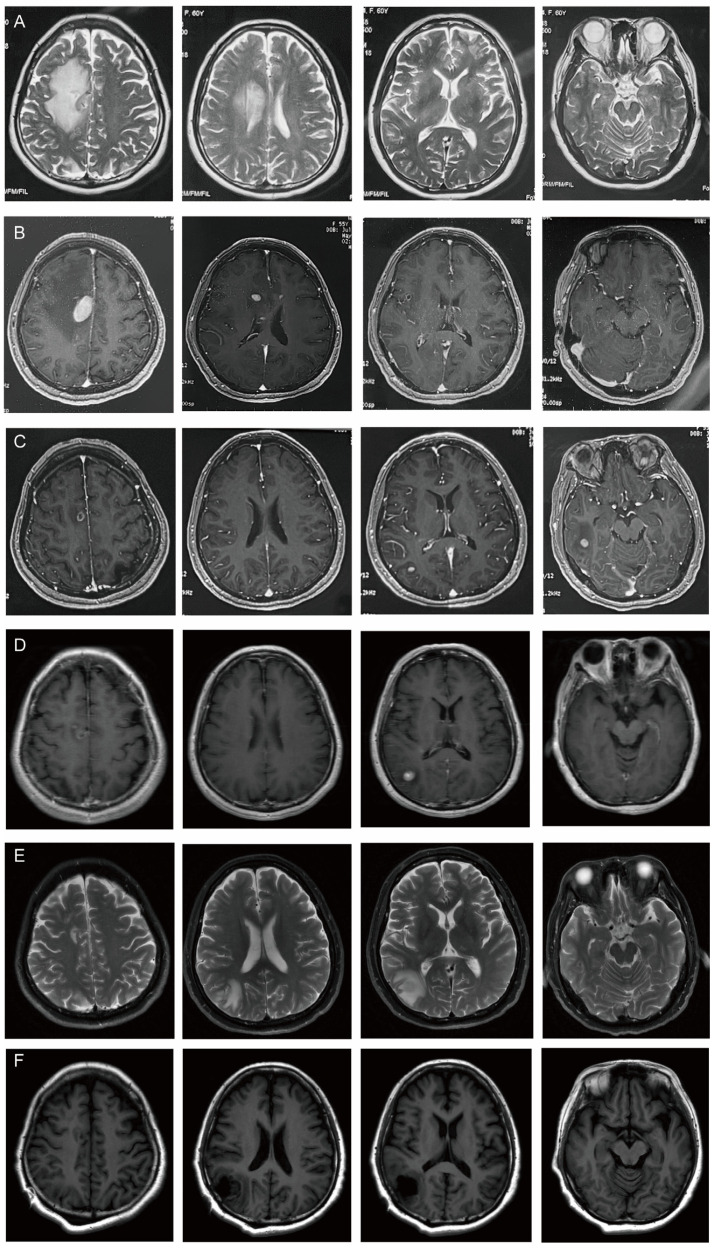
MRI images of patient’s brain during the diagnosis and treatment. Lesions in different parts are shown from left to right: the right centrum semiovale, the top of the lateral ventricle, right occipital lobe, right temporal lobe. (**A**) 18 April; (**B**) 6 May; (**C**) 24 June; (**D**) 9 July; (**E**) 22 July; (**F**) 24 July.

A cervico-thoracic CT scan was performed on 18 April 2024, which showed small lymph nodes in both necks and patchy low-density shadows on the right side of the brain. Cranial enhancement MRI showed a nodular abnormal signal next to the anterior horn of the lateral ventricle in the frontal lobe area of the right side of the brain; the large one was about 1.7 × 1.0 cm, with clear boundary, isointense on T1WI and T2WI, hyperintensity on FLAIR and DWI. It was obviously heterogeneously enhanced, and there were patchy non-enhancement areas around it. A metastatic tumor with surrounding edema was considered. Color Doppler ultrasound of the thyroid and cervical lymph nodes suggested that the echo of the left lobe of the thyroid was uneven. Hashimoto’s thyroiditis was considered. No obvious enlarged lymph nodes were found in the bilateral neck. On 22 April 2024, AFP was 8.200 ng/mL (reference range 0.00–7.00) and CA72–4 was 15.74 IU/mL (reference range 0–6.90). The MRI images of the patient’s diagnosis and treatment are shown in Figure 2.

**Figure 3:**
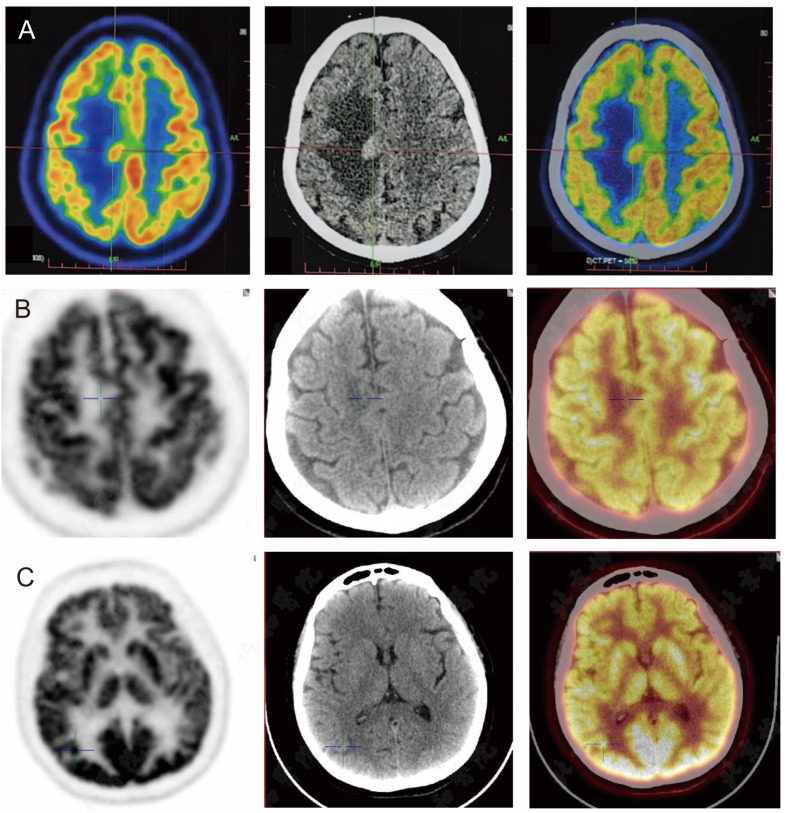
PET/CT images of the patient’s brain during the diagnosis and treatment. (**A**) 22 April: a hypermetabolic focus with peripheral edema in the right centrum semiovale (SUVmax 14.3); (**B**) 8 July: nodules of centrum semiovale in the right, metabolically lower than the cortex; (**C**) 8 July: nodules of increased metabolism in the right occipital lobe (about 0.7 cm, SUVmax17.4).

**Figure 4:**
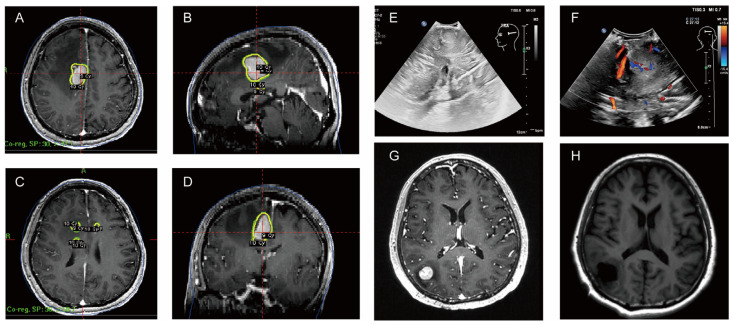
Treatment diagram. (**A**–**D**) Radiation therapy sites. (**E**,**F**) Ultrasound of intraoperative resection of the lesion. (**G**) Preoperative enhanced MRI shows right occipital lobe occupancy on 22 July 2024. (**H**) Postoperative MRI showed satisfactory resection on 24 July 2024.

On 22 April 2024, the patient underwent PET/CT, which suggested a hypermetabolic lesion with peripheral edema in the right centrum semiovale (SUVmax 14.3) (Figure 3A). On 6 May 2024, contrast-enhanced MRI of the head showed intracranial multiple space-occupying lesions, which were located in the right frontal lobe and the top of the lateral ventricle (Figure 3B). Thyroid cancer metastasis was considered in another hospital, and the patient received gamma knife radiotherapy for right frontal lobe and lateral ventricle lesions on the same day. Considering that this patient had a large lesion and some small lesions nearby, with relatively heavy odema, the treatment should generally be staged to avoid too heavy postoperative reactions. Generally, the tumor will shrink in about four weeks after the first treatment, and then it will be safer to have a second treatment. The prescription dose was 10 Gy for the periphery of the target and 20 Gy for the center (Figure 4A–D) for the first treatment. After the first treatment, the patient’s symptoms of poor left limb movement were significantly improved. On 24 June 2024, the patient had a follow-up cranial contrast-enhanced MRI, which showed that the lesions in the right frontal lobe and the top of the lateral ventricle had decreased, but new lesions appeared in the right temporal lobe and occipital lobe.

For further evaluation, the patient had a repeat cranial contrast-enhanced MRI on 9 July 2024 at our hospital, which showed that the lesion in the right temporal lobe was smaller than before, but the lesion in the right occipital lobe was larger than the previous one. PET/CT examination on 8 July 2024 showed a nodule (about 0.7 cm, SUVmax 17.4) with hypermetabolism in the right occipital lobe, which was considered to be malignant (Figure 3B). The right centrum semiovale and right temporal lobe nodule showed lower metabolism than the cortex, and malignancy cannot be excluded (Figure 3C). No abnormal metabolic increase was observed in the thyroid operative area. Navigation localization and head-enhanced MRI were completed on 22 July, which showed multiple intracranial space-occupying lesions. The right occipital space-occupying lesions were enlarged, obviously enhanced, and accompanied by edema compared with the previous radiograph of our hospital (9 July 2024).

As for thyroglobulin (TG), on 16 June 2021, TG was 0.06 ng/mL (reference range 3.5–77) and on 16 September 2021, TG was 0.32 ng/mL (reference range 3.5–77). On 20 June 2024, TG was 0.55 ng/mL (reference range 3.5–77) and anti-thyroglobulin antibodies (TGAb) were 9.72 IU/mL (reference range < 4.11).

### 2.4. Course of Treatment

In view of the new imaging findings, the patient underwent surgical resection of the right occipital lobe lesion in our hospital (Figure 4E,F) and the postoperative head MRI was conducted on 24 July. Postoperative pathology showed a malignant tumor. Combined with immunohistochemistry and in situ hybridization results, it was considered to be an aggressive B-cell non-Hodgkin’s lymphoma with a preference for diffuse large B-cell lymphoma (Hans typing: germinal center B-cell subtype), with dual expression of CMYC and Bcl-2 protein levels. Immunohistochemical results were as follows: AE1/AE3(−), CK17(−), TTF-1(−), Thy(−), Ki-67(90%), PAX-8(+), Syn(−), CgA(−), INSM-1(−), Calcitonin(−), oligo-2(−), GFAP(−), Be-2(90%, +), Bcl-6(90%+), CD3(partial+), CD5(partial+), CD10(+), CD20(++), CD30(Ki-1)(−), C-MYC(50%,+), Mum-1(90%,+), P53(partial+), CD19(++). In situ hybridization result: EBER ISH(−).

After surgery, the patient was referred to the Department of Hematology for further consultation and diagnosis with primary central nervous system diffuse large B-cell lymphoma (GCB subtype, DE+, DH to be investigated, IELSG score 1, low risk). The treatment regimen was adjusted to a C1-POR regimen (pomalidomide 4 mg qd d1-14 + obrutinib 150 mg qd d1-21 + rituximab 600 mg iv d1). The cerebrospinal fluid cytology and protein of the patient were abnormal, and lymphoma involving the leptomeninges was considered. Lumbar puncture and intrathecal injection of Arc-C 50 mg, Dex 5 mg, and MTX 10 mg were performed.

### 2.5. Follow-Up and Prognosis

Post-treatment follow-up showed that the patient had a partial response to treatment, and imaging showed some lesions had shrunk. The patient remains under close observation with ongoing evaluation to monitor disease progression and response to treatment.

## 3. Discussion

### 3.1. Diagnostic Challenges

In this case, the patient had a history of thyroid cancer, and after the presentation of new neurological symptoms with a heterogeneous enhancement pattern, brain metastases were first considered. The PET-CT of 22 April 2024 showed hypermetabolic lesions with a large area of peripheral edema in the right centrum semiovale, which was consistent with metastatic tumor changes. However, there were atypical brain metastasis features, such as hypermetabolic focus in the deep white matter and paraventricular regions, which were consistent with the imaging findings of PCNSL. After the first stage of gamma knife treatment, the patient’s lesions reduced significantly and the symptoms improved well, which were not the typical changes of brain metastases. The differential diagnosis of such lesions includes brain metastases, glioma, and infectious or inflammatory processes. However, because of the patient’s past medical history, brain metastases remain a priority diagnosis.

Brain metastases only of thyroid cancer are indeed rare, but there have been cases of single brain metastases reported [16,17]. PCNSL is also rare after surgery of thyroid cancer without chemoradiotherapy. The TGAb of this patient were consistently high, and TG tended to rise after thyroid cancer surgery. Preoperative TG levels were unknown. Patients with normal or low serum TG before surgery would not show elevated serum TG at disease recurrence [18]. Also, there is a possibility of transforming a differentiated thyroid cancer to an anaplastic variety over time. In such a case, TG will not rise in the presence of recurrence [19]. Furthermore, the level of TGAb affects the judgement of recurrence and metastasis of thyroid cancer by TG levels. The diagnostic challenge in this case was the difficulty in clearly differentiating PCNSL from brain metastases on imaging, and the diagnosis of PCNSL was ultimately confirmed by surgical resection of the intracranial lesions and pathology. This suggests the importance of obtaining a histological diagnosis when imaging findings are atypical or inconclusive.

### 3.2. Treatment and Prognosis of PCNSL

The treatment of PCNSL differs significantly from that of brain metastases, making it important to differentiate between the two. PCNSL is usually treated for induction with high-dose methotrexate-based combinations and consolidation with high-dose chemotherapy combined with autologous hematopoietic stem cell transplantation (HDC-ASCT), non-myeloablative chemotherapy alone, and whole-brain radiotherapy at reduced doses (WBRT) [12]. The treatment options for PCNSL have increased in recent years with advances in targeted therapy and immunotherapy. Surgery in the management of PCNSL is usually limited to biopsy to confirm the diagnosis, as the tumor is usually diffuse and cannot be completely removed [20]. The prognosis for PCNSL is usually poor, with a median overall survival of 25.3 months in immunocompetent patients [21]. In contrast, treatment options for brain metastases are more diverse and may include surgical resection, radiation therapy (e.g., gamma knife), and targeted or whole-brain radiotherapy. In this case, the patient was initially treated with gamma knife, based on the assumption of brain metastases. However, after postoperative pathology confirmed the diagnosis of PCNSL, the treatment plan was adjusted to chemotherapy.

### 3.3. Analysis of Causes

The coexistence of thyroid cancer and PCNSL is extremely rare and has been poorly reported in the literature. Some of the literature has reported thyroid cancer following treatment for malignant tumors, such as non-Hodgkin’s lymphoma, and the reports were largely limited to children. It was thought that they may be related to chemotherapy and radiotherapy for the first tumor [22]. Thyroid cancer secondary to Hodgkin’s lymphoma but not PCNSL was reported in a large study; it was thought to be related to radiotherapy and chemotherapy [23]. PCNSL formation may occur as a result of first malignancy treatment and reflects an inherent predisposition to undetermined neoplastic transformation [24]. This patient did not undergo radiotherapy after surgery for thyroid cancer, which traditionally may be considered to contribute to the risk of developing lymphoma, but was then found to have PCNSL, which may involve multiple mechanisms such as immune dysregulation, chronic inflammation, and immune escape. Firstly, the patient’s thyroglobulin antibodies and ultrasound findings were consistent with a diagnosis of Hashimoto’s thyroid. Hashimoto thyroiditis is a common autoimmune thyroid disease that has been widely associated with the development of thyroid cancer [25,26]. The chronic inflammatory environment in Hashimoto’s thyroiditis may provide the basis for the abnormal proliferation and malignant transformation of thyroid cells. Patients with Hashimoto’s thyroiditis usually have elevated levels of IL-6, and the sustained high expression of this pro-inflammatory cytokine not only promotes the local inflammatory response in the thyroid gland but also may promote the occurrence and progression of tumors by activating the JAK/STAT3 signaling pathway [27], which is consistent with the preoperative laboratory findings of this patient with elevated IL-6 (12.28 pg/mL, reference value ≤ 5.30 pg/mL). Meanwhile, the development of PCNSL has been associated with autoimmune diseases such as systemic lupus erythematosus, polyarteritis nodosa, autoimmune hepatitis, myasthenia gravis, uveitis, and others [28]. The most important risk factor for primary thyroid lymphoma (PTL) has been reported to be chronic autoimmune lymphocytic thyroiditis (Hashimoto’s thyroiditis), which is associated with more than 90% of PTL [29]. Thus, Hashimoto thyroiditis, as an autoimmune disease, may also increase the incidence of PCNSL. Furthermore, in addition, high levels of IL-6 may also impair the body’s anti-tumor immune surveillance function by inhibiting the activity of cytotoxic T cells, such as CD8+ T cells [30]. A decrease in the number of CD8+ T cells found in this patient’s preoperative TB cell subpopulation examination (9.6%, reference value 19.0–48.0%) further indicates a weakening of immune surveillance. CD8+ T cells, as effector T cells, are responsible for recognizing and destroying tumor cells, but their weakened function may lead to the immune escape of tumor cells. With reduced CD8+ T cell activity, patients may have less effective immune surveillance of thyroid cancer and lymphoma cells, allowing for the simultaneous development of both tumors. Therefore, chronic inflammation in the context of Hashimoto’s thyroiditis, an abnormal immune microenvironment caused by elevated IL-6, and decreased CD8+ T cell function may jointly lead to the coexistence of thyroid cancer and PCNSL.

### 3.4. Steps for Clinicians Managing

When intracranial lesions are detected after thyroid cancer surgery, the patient should be thoroughly examined. Thyroid and neck ultrasound, thyroid endocrine examination, including FT3, FT4, T3, T4, TSH, TG, TGAb, and TPOAb; cervical and chest CT, and enhanced MRI for intracranial lesions should be performed. If necessary, iodine scintigraphy or PET/CT can be used to assess the possibility of systemic metastasis or recurrence. For cases that cannot be confirmed, multi-disciplinary discussions involving neurosurgery, thyroid surgery, endocrinology, radiotherapy, radiology, and ultrasound could be helpful. For cases that remain inconclusive, surgery or biopsy to obtain pathology is the gold standard for diagnosis.

### 3.5. Research Innovations

First, this case report provides the first detailed analysis of the coexistence of thyroid cancer and primary central nervous system lymphoma (PCNSL), revealing a potential link between chronic inflammation associated with Hashimoto’s thyroiditis and tumorigenesis. Second, this article provides insight into how alterations in the patient’s immune microenvironment may have led to tumor coexistence, providing a new perspective on tumor immunology. Moreover, the diagnostic course of this case demonstrates the importance of imaging versus pathology, especially in the context of a new lesion in the nervous system, highlighting the importance of histological examination. This case report enriches the existing literature and suggests the need for clinicians to be on high alert when dealing with similar patients.

## 4. Conclusions

The metachronous malignancy found after surgery of thyroid cancer in this case demonstrates the diagnostic and therapeutic challenges of such a rare condition, emphasizes the importance of maintaining a broad differential diagnostic mindset when new neurological symptoms are present in a patient with cancer, and underscores the importance of pathological confirmation of the diagnosis for the development of appropriate treatment strategies. Future research is needed to further understand the pathogenesis of PCNSL in the context of thyroid cancer and to optimize therapeutic strategies for this rare but challenging clinical condition.

## Figures and Tables

**Table 1:** The course of the patient’s diagnosis and treatment.

Time	Diagnosis and Treatment
December 2019	Thyroid isthmus and by lobe resection
	Papillary thyroid carcinoma (T1N0M0) with BRAF V600E mutation
April 2024	Unfavorable movement of left limb, dizziness
18 April–6 May 2024	CT (4.18): small lymph nodes in both necks, patchy hypodense shadow in the right side of the brain after right lobectomy of the thyroid gland
	MRI (4.18): nodular abnormal signals in the right frontal lobe of the brain next to the anterior horn of the lateral ventricle. It was considered to be metastatic tumor accompanied by peripheral odema.
	Ultrasound of the thyroid gland (4.18): heterogeneous echogenicity of the left lobe of the thyroid gland—Hashimoto’s thyroiditis perhaps
	PET/CT (4.22): hypermetabolic foci with peripheral odema in the right centrum semiovale; uneven metabolism in left lobe glands, multiple slightly hypermetabolic lymph nodes in neck and mediastinum bilaterally
6 May 2024	MRI (5.6): multiple intracranial lesions in the right frontal lobe and top of the lateral ventricles.
	gamma knife radiation therapy
24 June–9 July 2024	MRI (6.24): The lesions in the right frontal lobe and the top of the lateral ventricle reduced, but new lesions appeared in the right temporal lobe and occipital lobe.
	MRI (7.9): Right temporal lobe lesions were smaller, but right occipital lobe lesions were larger.
	PET/CT (7.8): A metabolically increased nodule in the right occipital lobe (SUVmax 17.4); a nodule in the right centrum semiovale and the right subject lobe, with metabolism lower than that of the cortex
July 2024	Surgical resection
	Pathology: considered aggressive B-cell non-Hodgkin’s lymphoma
August 2024	Referred to the haematology department, and after further refinement of investigations, diagnosis of primary central diffuse large B-cell lymphoma (GCB subtype, DE+, DH to be investigated, IELSG score 1, low risk)
	A C1-POR regimen

## Data Availability

Data are contained within the article.
